# Personalized Medicine for Nervous System Manifestations of von Hippel–Lindau Disease

**DOI:** 10.3389/fsurg.2016.00039

**Published:** 2016-06-30

**Authors:** Victoria Schunemann, Kristin Huntoon, Russell R. Lonser

**Affiliations:** ^1^Department of Neurological Surgery, Ohio State University Wexner Medical Center, Columbus, OH, USA

**Keywords:** von Hippel–Lindau, personalized medicine, hemangioblastoma, histone deacetylase inhibitor, endolymphatic sac tumor

## Abstract

von Hippel–Lindau disease (VHL) is a familial neoplasia syndrome associated with multisystem tumor development. Depending on tumor type and location, current treatments for VHL-associated tumors can include a combination of chemotherapy, radiation therapy, and/or surgery. Central nervous system (CNS) manifestations of VHL include craniospinal hemangioblastomas and endolymphatic sac tumors (ELSTs). While the first-line treatment for both types of VHL-associated CNS tumors is surgery, the indications for treatment are patient specific and different for each tumor type. Although early sign/symptom formation is the primary indication for resection of craniospinal hemangioblastomas, radiographic discovery (asymptomatic and symptomatic) of ELSTs can be an indication for resection of ELSTs in VHL patients. Recently, research has revealed that specific *VHL* germline mutations may permit targeted medical treatments of not only CNS manifestations of VHL-associated tumors but also visceral tumors. Specifically, missense mutations can result in the translation of functional VHL protein (pVHL) that is rapidly degraded resulting in functional loss of the pVHL, and inhibitors of pVHL degradation may slow protein degradation and restore pVHL function. Emerging research will investigate the safety and practicality of using potential targeted therapies.

## Introduction

**Figure 1:**
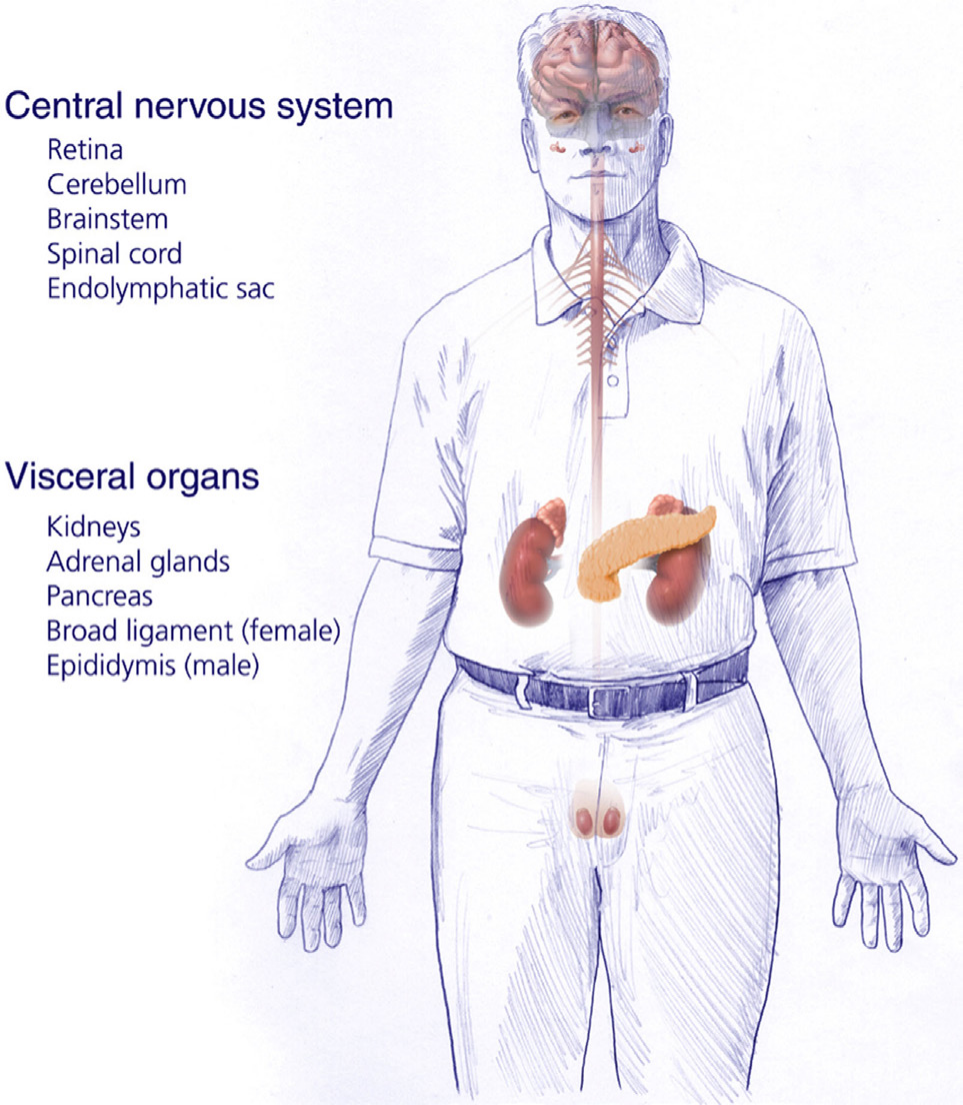
**Central nervous system and systemic locations and types of tumors of patients harboring germline protein VHL mutations and von Hippel–Lindau disease [adapted from Lonser et al. (7)]**.

von Hippel–Lindau disease (VHL) is an autosomal dominant inherited genetic disorder caused by a germline mutation of chromosome 3 (*VHL gene*). Patients affected with VHL develop multiple central nervous system (CNS) lesions, including retinal and craniospinal hemangioblastomas, as well as endolymphatic sac tumors (ELSTs). Visceral VHL-associated lesions frequently include renal cell carcinomas, renal cysts, pheochromocytomas, extra-adrenal paragangliomas, pancreatic microcystic adenomas, pancreatic cysts, and pancreatic neuroendocrine tumors, as well as cystadenomas of the epididymis and broad ligament (1–3) (Figure 1). VHL has an incidence of approximately 1 in 36,000–39,000 live births (4, 5). Penetrance is nearly complete and most (over 90%) patients display evidence of the disease by the age of 65 years (6).

Treatment of VHL is complex due to the multisystem involvement, the location of tumors, and the immense variability of symptoms that can be produced related to various tumor locations and systems involved. Recent studies have elucidated new germline-based targets for treatment. Existing treatments include combinations of chemotherapy, radiation therapy, and surgical resection. We describe the current personalized management of the CNS manifestations of VHL and the potential putative therapeutics that are based on germline mutation types.

## Genetics and Pathogenesis

Mutations in the *VHL gene* lead to the development of the manifestations of VHL. The *VHL gene* is located on the short arm of chromosome 3 (3p) and is a tumor suppressor gene (8). Germline mutations of *VHL* account for more than 95% of the patients affected by VHL (5% have somatic inactivation of the *VHL* gene in sporadically occurring hemangioblastomas and renal cell carcinomas) (9). VHL patients inherit a *VHL* germline mutation from the VHL-affected parent and a normal (wild-type) gene from the non-affected parent. Tumorigenesis occurs when the wild-type *VHL* allele is inactivated (loss of heterozygosity) in certain susceptible target organs that include the viscera (kidneys, pancreas, adrenal glands, and adnexal organs), as well as the CNS (7).

**Figure 2:**
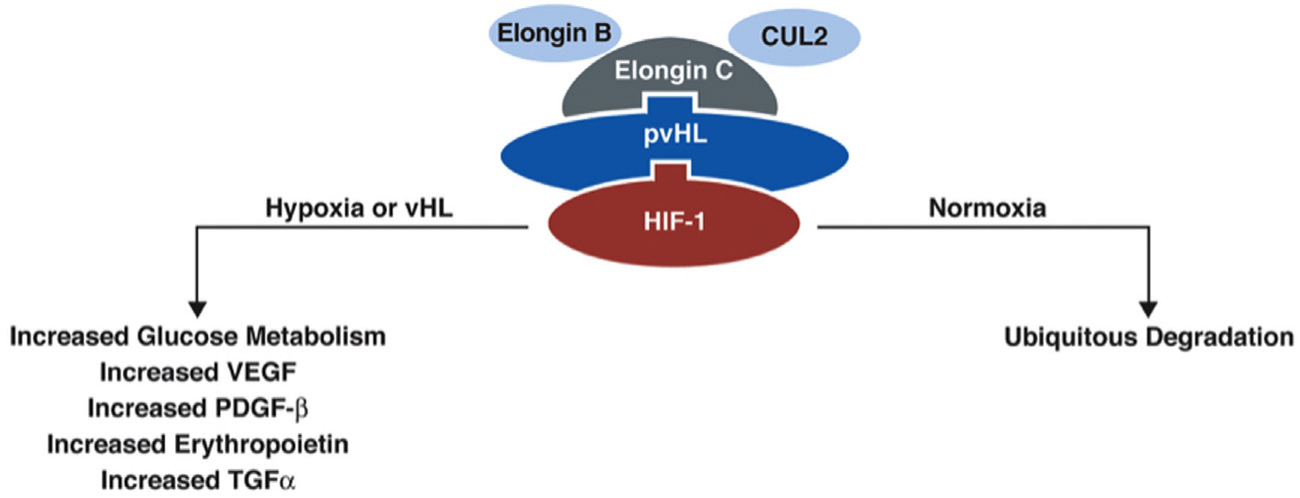
**Function of protein VHL in the proteasome**. pVHL is thought to function as an E3 ubiquitin ligase in the proteasome complex and bind HIF-1α, which results in ubiquitination of HIF-1α and leads to degradation. In normoxic conditions, HIF-1α is degraded, but in conditions of hypoxia, HIF-1α is upregulated. In the absence of pVHL, HIF-1α is not ubiquitinated and degraded [adapted from Lonser et al. (7)].

The *VHL gene* encodes VHL protein (pVHL), a protein that is part of the E3 ubiquitin ligase, which is involved in proteasomal degradation. It targets hypoxia inducible factor (HIF)-1/2α (10) transcription factors that are activated in hypoxic conditions to upregulate genes, including vascular endothelial growth factor (VEGF), transcription growth factor (TGF), erythropoietin (EPO), EPO receptor, transferrin, and angiopoietin (11). These factors are involved in angiogenesis, erythropoiesis, cell proliferation, and/or tumorigenesis/metastasis. HIF-2α is a known oncogene that contributes to cell proliferation and tumorigenesis (11). pVHL participates in degradation of HIF-1/2-α by binding the transcription factors to the proteasome complex (Figure 2). When the *VHL gene* is mutated and its function is reduced/lost, HIF-1/2α is upregulated (even in the absence of hypoxic conditions) due to its reduced degradation by the VHL ubiquitin–proteasome complex (7).

Multiple VHL germline mutations have been discovered, ranging from deletions to missense mutations. Germline VHL missense mutations are the most common and underlie 60–70% of all VHL-associated mutations (4). Recent studies have shown that the proteins translated from the missense mutated *VHL gene* are highly unstable and rapidly degraded (10), but retain the functional capacity of wild-type protein. Consequently, treatment strategies that extend the half-life of pVHL in this circumstance could lead to normalization (reversal) of VHL-related pathobiologic features.

## VHL-Associated Tumors

### Hemangioblastomas

**Figure 3:**
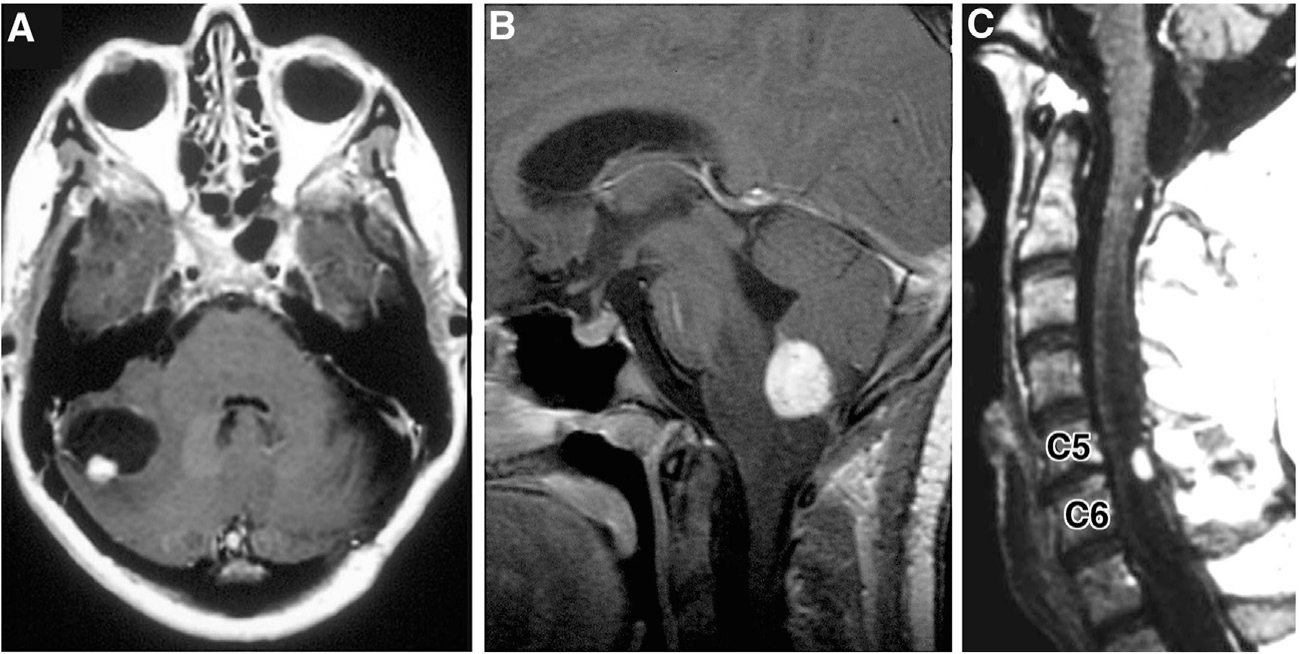
**Radiographic images of hemangioblastomas**. **(A)** Axial, contrasted, T1-weighted MRI showing cerebellar hemangiolastoma with contrast enhancing mural nodule and peritumoral cyst. **(B)** Sagittal, contrasted, T1-weighted MRI revealing contrast enhancing medullary hemangioblastoma with surrounding vasogenic edema. **(C)** Sagittal, contrasted, T1-weighted MRI with contrast enhancing posterior/dorsal hemangioblastoma with associated syrinx [adapted from Lonser et al. (7)].

Hemangioblastomas are highly vascular tumors that arise in the CNS. They are the most common tumor presentation of VHL patients. Previously, studies have estimated that 60–90% of VHL patients will develop multiple hemangioblastomas in their lifetime (12, 13). Cerebellar lesions are the most common, followed by spinal cord, brainstem, and supratentorial tumors (Figure 3) (3, 9). CNS hemangioblastomas are histologically benign but cause a multitude of symptoms and can result in death depending on their location and size. Symptomatic CNS hemangioblastomas are most frequently associated with peritumoral cysts, although symptoms can be caused by solid tumors and are location dependent (1, 14, 15).

Recent natural history studies have provided a better understanding of the growth and development of hemangioblastomas in VHL. We prospectively studied 250 VHL disease patients with a total of 1921 CNS hemangioblastomas (9). At the end of the study, mean number of craniospinal hemangioblastomas had increased from 7 to 8 per person over a mean follow up of 6.9 years (new hemangioblastoma development was inversely associated with age). When observed out to 5 years, 49% of known hemangioblastomas progressed in size in a linear, saltatory, or exponential pattern. Brainstem and cerebellar hemangioblastomas grew significantly faster than the spinal or cauda equina hemangioblastomas. Male sex was associated with a significantly faster growth rate than females. Most VHL patients will develop multiple hemangioblastomas over time. Because hemangioblastomas grow at different rates, in multiple locations, and exhibit irregular growth patterns, symptom formation can be unpredictable. Surgical resection is often reserved until the first onset of signs/symptoms that correlate with the location of the hemangioblastoma. This management paradigm attempts to ensure that unnecessary surgical intervention and associated possible complications are avoided. This can often result in maintenance of baseline neurologic function for most patients (9).

Peritumoral cysts are a frequent cause of hemangioblastoma-associated signs/symptoms, and they form by a plasma ultrafiltrate passing into tissue surrounding the hemangioblastoma from permeable tumor blood vessels (1). Previously, we prospectively followed 225 patients with VHL disease, of which 132 patients had 292 peritumoral cysts (14). Approximately 75% of peritumoral cysts progressed within 3 years. Cysts grew faster if located in the cerebellum, in patients under 35 years of age, and if they were associated with symptoms. Peritumoral cysts appeared to grow in three patterns, including saltatory (phases of growth followed by stability), linear, or exponential. Overall, a majority of the cysts grew in a saltatory manner (41.7%). However, of the 60 symptomatic peritumoral cysts, 45% grew exponentially (9). Risk of an increased total number of tumors was significantly associated with a partial deletion germline mutation and male sex, while new cyst development was associated with a greater number of cysts at the time of initial evaluation for the study and age younger than 35 years.

Radiation therapy [most frequently stereotactic radiosurgery (SRS)] has been utilized for treatment of CNS hemangioblastomas in VHL. Asthagiri and colleagues (16) prospectively evaluated the effect of SRS on craniospinal hemangioblastomas in 20 VHL patients (11 symptomatic, 9 asymptomatic) with 44 hemangioblastomas. Fourteen tumors (32%) progressed after SRS treatment, and four of these tumors required surgical resection. Local control rates decreased over time with 91, 83, 70, 61, and 51% at 2, 5, 8, 10, and 15 years, respectively, and were similar to rates of progression in untreated hemangioblastomas (9). These data indicate that SRS should be reserved for treating hemangioblastomas that are not surgically resectable or in patients who cannot tolerate surgical resection (16).

## Endolymphatic Sac Tumors

Endolymphatic sac tumors are vascular, low-grade papillary adenocarcinomas affecting up to 11% of VHL patients. Mean age of diagnosis is 22 years, and bilateral ELSTs are found in approximately 30% of patients with VHL (17). The majority of the patients had associated audiovestibular symptoms, including sensorineural hearing loss (84% of ears), tinnitus (73%), and vertigo (68%) that did not correlate with tumor size (18). The audiovestibular findings associated with ELSTs are thought to be due to intralabyrinthine hemorrhage, endolymphatic hydrops, and/or direct invasion of the otic capsule by tumor (19). Sudden hearing loss (43%) has been correlated with intralabyrinthine hemorrhage. Gradual hearing loss (47%) is most often related to endolymphatic hydrops.

Regular screening of VHL patients for ELSTs is recommended, with surgical intervention in selected patients before morbidity develops. Surgery is curative for completely excised tumors. Kim and colleagues found that hearing was stabilized postoperatively in 90% of patients after ELST resection (18). Current indications for ELST resection in VHL patients include imaging evidence of an ELST with serviceable hearing (and/or audiovestibular signs/symptoms), evidence of ELST-associated intralabyrinthine hemorrhage, ELST-associated hydrops, or mass effect by the ELST (19, 20). Contrast-enhanced delayed FLAIR MRI has been found to be an efficacious, non-invasive method of detecting ELST-associated hydrops (21). The role of adjuvant therapy, including chemotherapy, fractionated radiotherapy, or stereotactic radiosurgery, is not established.

## Emerging Targets for Treatment of VHL

Recent investigations into the pathogenesis of VHL tumors have revealed new potential targets for treatment. Metelo and colleagues (22) studied VHL models in zebrafish using vhl^−/−^ embryos. Treatment with HIF-2α inhibitors decreased expression of HIF-2α targeted genes. The effect was dose dependent in these studies. It improved new angiogenic sprouting that was seen in vhl^−/−^ embryos and returned abnormal cardiac function to baseline, suggesting that HIF-2α could lead to potential targeted treatments for systemic VHL tumors.

The most frequent mutations in VHL are missense mutations. VHL patients that harbor a missense germline mutation have a quantitative reduction of missense mutant VHL protein (pVHL), but still maintain physiologic pVHL mRNA expression. Recent data indicate that mutant pVHL is highly unstable and is quickly degraded after translation. Interestingly, missense mutant pVHL retains its E3 ligase function, including HIF degradation. The premature pVHL degradation is due to misfolding and imbalance of chaperonin binding (23).

Histone deacetylase inhibitors (HDACis) can modulate the pVHL degradation pathway by inhibiting the HDAC6–Hsp90 chaperone axis, stabilizing pVHL, and restoring activity comparable to wild-type protein *in vitro* and in mouse VHL models. HDACi-mediated stabilization of missense pVHL significantly attenuates the growth of mouse VHL tumors (23). These findings provide direct insight into the pathobiology of VHL-associated tumors and elucidate a new treatment paradigm for personalized therapy in those individuals with missense VHL mutations.

## Personalized Approaches

von Hippel–Lindau disease is a complex and progressive process involving multisystem tumor formation. Individuals afflicted with VHL disease require a personalized approach for therapy, as tumors are neither uniform in their locations nor identical in their symptomatology. Currently, successful systemic treatments are lacking. The first choice of therapy for hemangioblastomas and ELSTs is surgery, and the decision to proceed with surgery is personalized to the individual. Surgery is reserved for hemangioblastomas based on symptom development and progression in tumor or cyst size. Surgery for ELSTs can also be based upon symptomatology, but indications also include radiographic findings of ELSTs and serviceable hearing. Care is taken to tailor the exact treatment to each individual patient and their tumor burden.

Recent studies revealed missense mutations in the *VHL gene* actually allow for transcription of the protein, albeit an unstable one that is rapidly degraded due to misfolding and chaperonin binding. The protein is then unable to function and degrade its target, HIF-2α as part of the proteasome. HDACis interfere with chaperonin pathways and, in result, stabilize the protein and allow return to function *in vitro* and in VHL mouse models. This offers a unique opportunity for a new treatment modality that in theory would be able to treat the underlying mechanism of tumorigenesis and affect the whole body, multiple systems, and possibly all of the differing tumor types in the body.

Further investigation and Phase I clinical trials would need to be conducted to assess the feasibility of developing HDACis as possible treatments. Currently, vorinostat, an HDACi, is undergoing Phase I trials for VHL patients with hemangioblastomas and missense mutations, trial #NCT02108002. With the potential of new therapies on the horizon, this could permit further treatment of the complex manifestations of VHL disease tailored to the individuals with this most common VHL germline mutation.

## Conclusion

von Hippel–Lindau disease is a complex disorder, and patients develop a wide constellation of symptoms related to the varying locations and types of tumors present. Currently, therapies are tailored toward the individual tumors and patient findings. Discovery of pVHL stabilization with use of HDACis in missense mutated pVHL provides a potential for new treatment directed toward the underlying mechanisms of tumorigenesis that could further tailor therapy toward the individual with VHL and offer a uniform treatment for various tumors associated with the disease.

## Author Contribution

All three authors participated in the research, composition, drafting, and editing of the manuscript.

## Conflict of Interest Statement

The authors declare that the research was conducted in the absence of any commercial or financial relationships that could be construed as a potential conflict of interest.
